# Crystal structure of polymeric bis­(3-amino-1*H*-pyrazole)­cadmium dibromide

**DOI:** 10.1107/S2056989023009751

**Published:** 2023-11-14

**Authors:** Iryna S. Kuzevanova, Oleksandr S. Vynohradov, Vadim A. Pavlenko, Sergey O. Malinkin, Sergiu Shova, Igor O. Fritsky, Maksym Seredyuk

**Affiliations:** a Department of General and Inorganic Chemistry, National Technical University of Ukraine "Igor Sikorsky Kyiv Polytechnic Institute", Peremogy Pr. 37, 03056, Kyiv, Ukraine; b Innovation Development Center ABN, Pirogov str. 2/37, 01030 Kyiv, Ukraine; c Department of Chemistry, Taras Shevchenko National University of Kyiv, Volodymyrska Street 64, Kyiv, 01601, Ukraine; d Department of Inorganic Polymers, "Petru Poni" Institute of Macromolecular Chemistry, Romanian Academy of Science, Aleea Grigore Ghica Voda 41-A, Iasi, 700487, Romania; University of Neuchâtel, Switzerland

**Keywords:** crystal structure, cadmium complex, coordination polymer, hydrogen bonding

## Abstract

The reaction of cadmium bromide tetra­hydrate with 3-amino­pyrazole (3-apz) in ethano­lic solution leads to tautomerization of the ligand and the formation of crystals of the title compound, *catena*-poly[[di­bromido­cadmium(II)]-bis­(μ-3-amino-1*H*-pyrazole)-κ^2^ *N* ^3^:*N* ^2^;κ^2^ *N* ^2^:*N* ^3^], [CdBr_2_(C_3_H_5_N_3_)_2_]_*n*_ or [CdBr_2_(3-apz)_2_]_*n*_. Its asymmetric unit consists of a half of a Cd^2+^ cation, a bromide anion and a 3-apz mol­ecule. The Cd^2+^ cations are coordinated by two bromide anions and two 3-apz ligands, generating *trans*-CdN_4_Br_2_ octa­hedra, which are linked into chains by pairs of the bridging ligands. In the crystal, the ligand mol­ecules and bromide anions of neighboring chains are linked through inter­chain hydrogen bonds into a two-dimensional network. The inter­molecular contacts were qu­anti­fied using Hirshfeld surface analysis and two-dimensional fingerprint plots, revealing the relative qu­anti­tative contributions of the weak inter­molecular contacts.

## Abstract 2

The title compound {[CdBr_2_(3-apz)_2_]}_*n*_ consists of a Cd^2+^ cation, bromide anions balancing the charge and bridging 3-amino­pyrazole (3-apz) mol­ecules. The Cd^2+^ cations are coordinated by two bromide anions and two 3-apz ligands, generating *trans*-CdN_4_Br_2_ octa­hedra, and are linked into chains by pairs of the bridging ligands. In the crystal, the 3-apz ligands and bromide anions of neighboring chains are linked through inter­chain hydrogen bonding into a two-dimensional supra­molecular network.

## 1. Chemical context

Inorganic–organic coordination polymers, an active field of investigation in chemistry, attract attention for their intriguing structures and applications. Inorganic components may introduce magnetic, optical, and mechanical attributes, while organic ligands offer versatility and luminescence. Combining these attributes yields novel materials with diverse properties such as catalysis, separation, luminescence, spin transition and more (Seredyuk *et al.*, 2015 ▸; Piñeiro-López *et al.*, 2021 ▸). The formation of a coordination polymer involves the self-assembly of organic ligands and metal ions, driven by strong and directional inter­actions such as metal–ligand coordination bonds, as well as weaker hydrogen bonds, π–π stacking, halogen–halogen, and C—H⋯*X* inter­actions (*X* = O, N, halogen, *etc*.). Engineering polymeric networks is a challenge that demands further exploration of metal–organic inter­actions.

The pyrazole is known to be a good linker to bind metal ions and play a key role in the design of new functional coordination polymers. It can serve as a monodentate ligand or upon deprotonation as a bridging ligand, effectively linking metal ions into polynuclear or polymeric moieties (Parshad *et al.*, 2024 ▸). We have discovered that 3-amino­pyrazole (3-apz) can form coordination polymers without the need to deprotonate the pyrazole moiety, due to the participation of the amino group in the coordination of the metal ion. Having an inter­est in polymeric complexes formed by bridging ligands (Piñeiro-López *et al.*, 2018 ▸, 2021 ▸; Seredyuk *et al.*, 2007 ▸), we report here on the coordination polymer of the apz ligand with a Cd^2+^ cation and Br^−^ anions as co-ligands. 
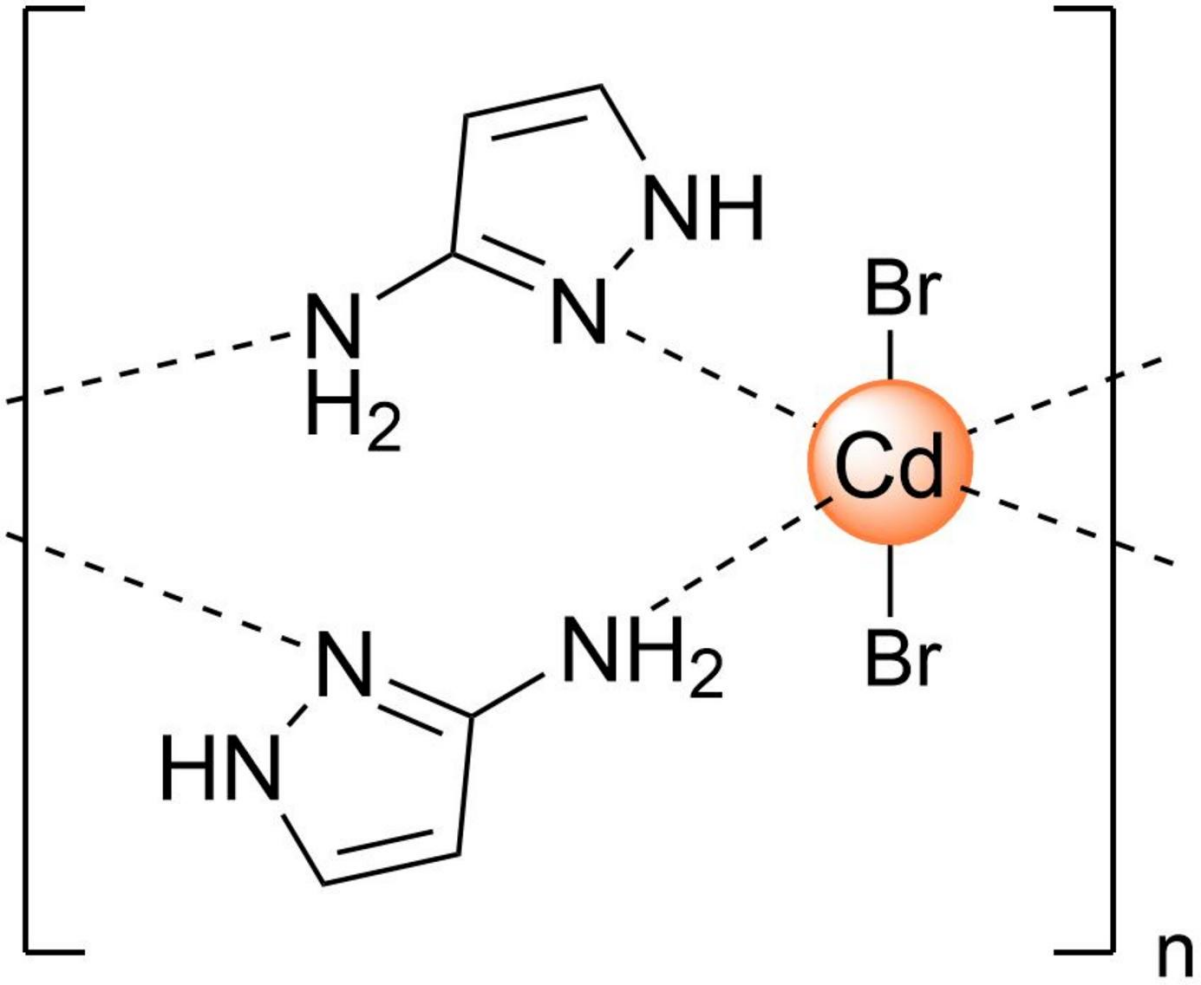


## 2. Structural commentary

**Figure 1:**
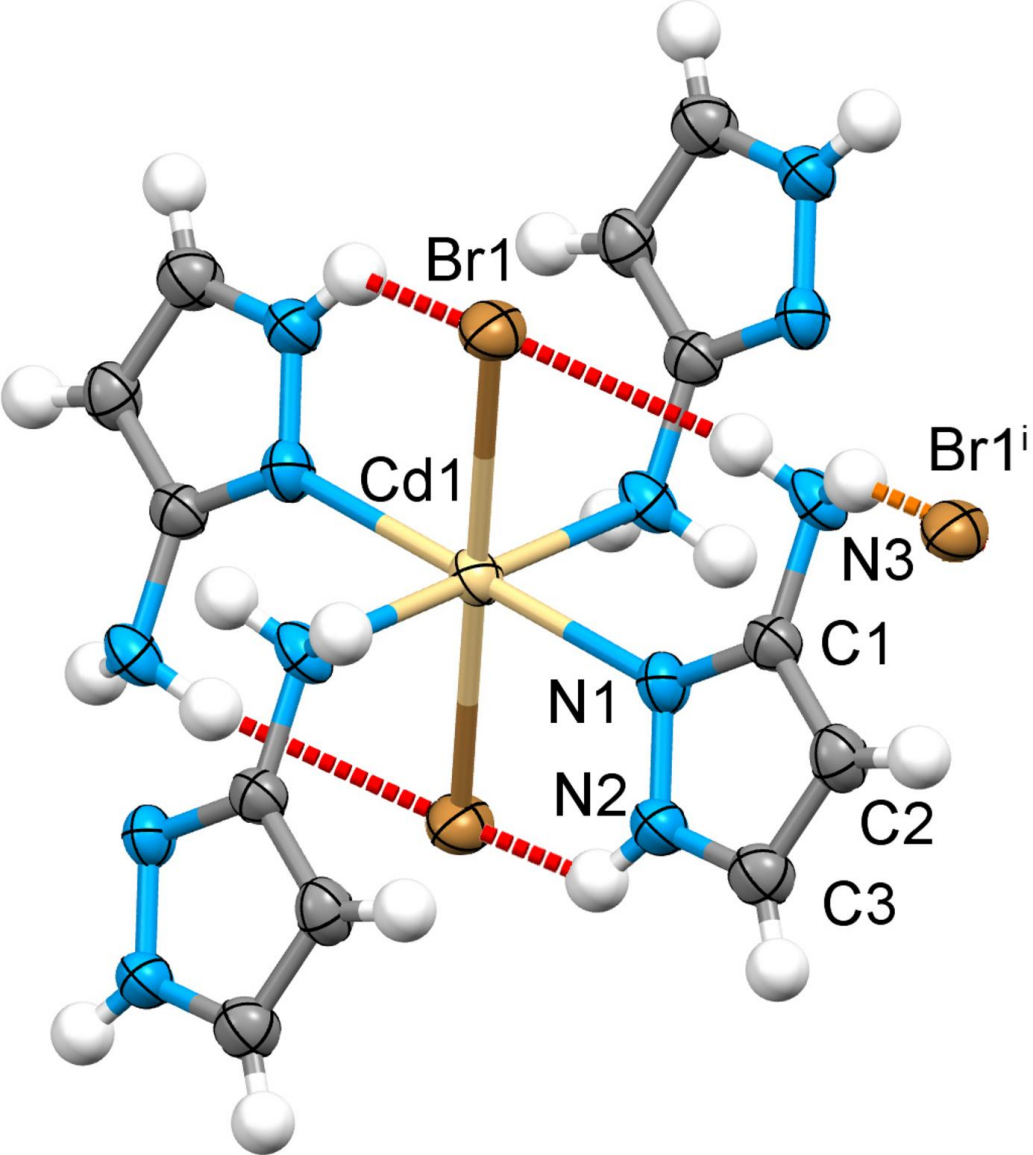
Crystal structure of the title compound with labelling and displacement ellipsoids drawn at the 50% probability level. The strong intra- and inter­chain N—H⋯Br hydrogen bonds are shown as dashed red and orange lines, respectively. Symmetry code: (i) 1 − *x*, −*y*, 1 − *z*.

The asymmetric unit comprises half of the monomeric neutral unit [Cd(3-apz)_2_Br_2_], which is composed of a Cd^2+^ cation, two 3-apz bridging ligands and two Br^−^ anions, balancing the charge (Fig. 1 ▸). The tautomerism of the ligand mol­ecule, which can inter­convert between 3- and 5-amino­pyrazole in solution, is blocked, and only the first form is observed in the structure. The coordination geometry around the central ion can be described as an elongated octa­hedron with the Br atoms being in axial positions [Cd—Br1 = 2.7379 (11) Å] and the amino nitro­gen atom of the 3-apz ligand [Cd—N1 = 2.358 (9) Å, Cd—N3 = 2.446 (9) Å] in the equatorial plane. The average trigonal distortion parameters *Σ* = Σ_1_^12^(|90 – *φ*_i_|), where *φ*_i_ is the angle N/Br—Cd—N′/Br′ (Drew *et al.*, 1995 ▸), and *Θ* = Σ_1_^24^(|60 – *θ*_i_|), where *θ*_i_ is the angle generated by superposition of two opposite faces of an octa­hedron (Chang *et al.*, 1990 ▸) are 34.6 and 112.4°, respectively. The values reveal a deviation of the coordination environment from an ideal octa­hedron (where *Σ* = *Θ* = 0). The calculated continuous shape measure (CShM) value relative to the ideal *O*_h_ symmetry is 0.578 (Kershaw Cook *et al.*, 2015 ▸). The volume of the [CdN_4_Br_2_] coordination polyhedron is equal to 20.952 Å^3^. The 3-apz ligand is close to planarity with a maximum deviation of 0.19 (1) Å from the plane of the pyrazole ring for the amino N3 atom.

## 3. Supra­molecular features

**Figure 2:**
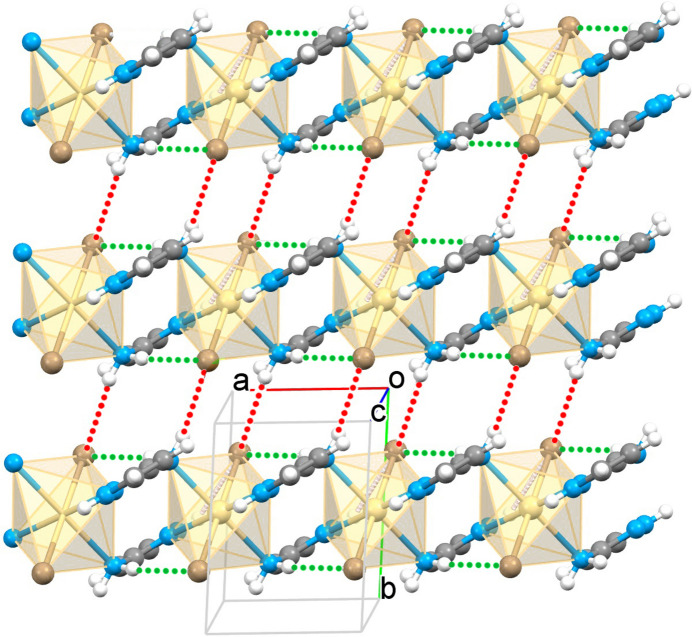
Fragment of the two-dimensional supra­molecular network formed by polymeric chains of {[CdBr_2_(3-apz)_2_]}_*n*_ with intra­chain hydrogen bonds (green dashed lines) linked by inter­chain hydrogen bonds (red dashed lines).

The [Cd(3-apz)_2_Br_2_] units are linked by alternating amino/pyrazole nitro­gen atoms of the 3-apz ligand to give an infinite one-dimensional linear chain propagating along the *a-*axis direction (Figs. 1 ▸ and 2 ▸). The Cd⋯Cd distance separated by 5-amino­pyrazole within the chain is 5.051 (1) Å. The N2 atom and one hydrogen of the NH_2_ groups of pyrazole are involved in inter­actions within the coordination chain, forming intra-chain hydrogen bonds with the Br atom (Table 1 ▸). The second hydrogen atom of the NH_2_ group forms a hydrogen bond with the Br atom of a neighboring chain. This inter­action expands the chains to a two-dimensional supra­molecular network (Fig. 2 ▸). The planes stack along the *c* axis with no inter­actions below the van der Waals radii.

## 4. Hirshfeld surface and two-dimensional fingerprint plots

**Figure 3:**
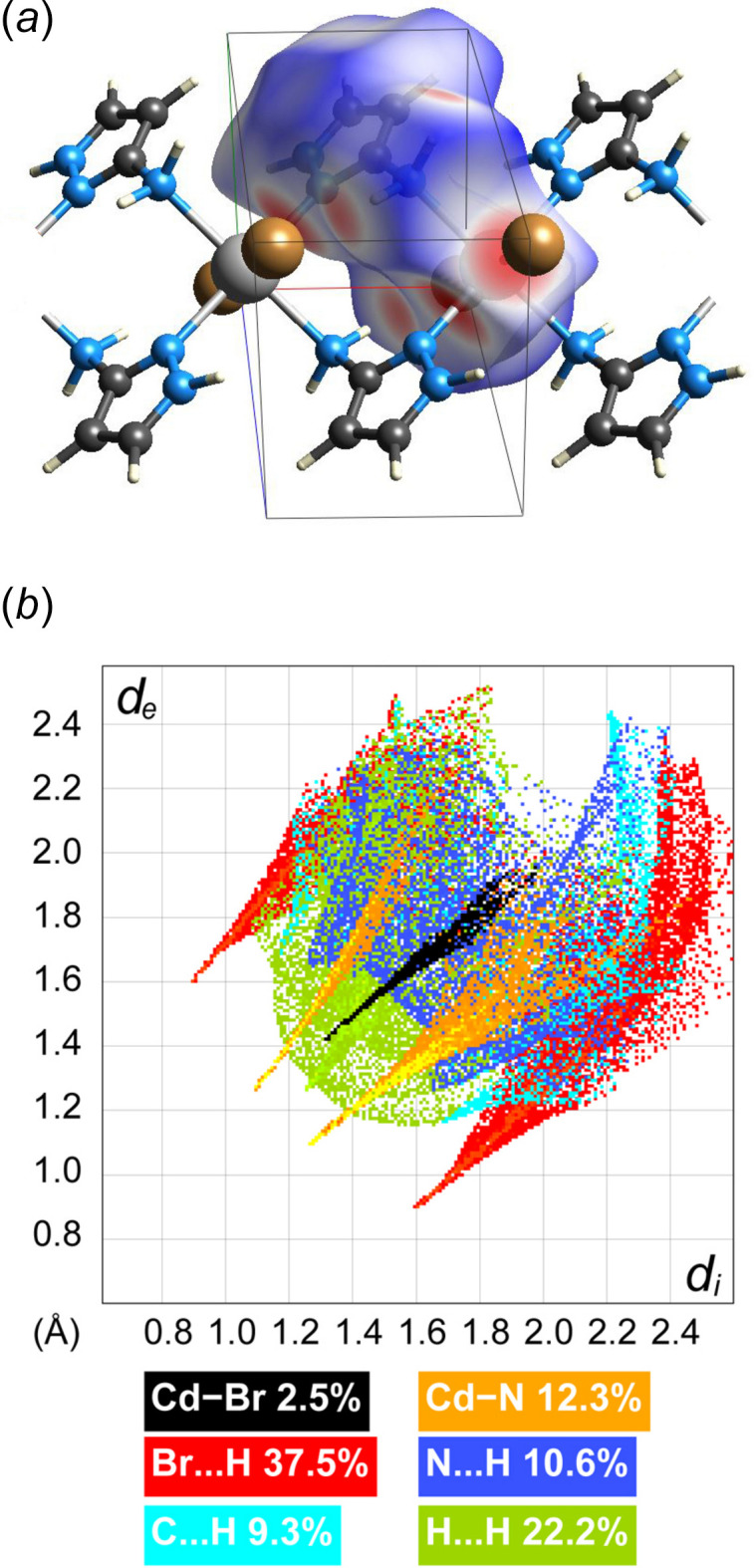
(*a*) A projection of *d*_norm_ mapped on the Hirshfeld surface onto a fragment of the polymeric chain in the asymmetric unit, visualizing intra- and inter­molecular inter­actions. Red/blue and white areas represent regions where contacts are shorter/longer than the sum and close to the sum of the van der Waals radii, respectively; (*b*) decomposition of the two-dimensional fingerprint plot into specific inter­actions.

Hirshfeld surface analysis was performed and the associated two-dimensional fingerprint plots were generated using *CrystalExplorer* (Spackman *et al.*, 2021 ▸), with a standard resolution of the three-dimensional *d*_norm_ surfaces plotted over a fixed colour scale of −0.4941 (red) to 1.0389 (blue) a.u. (Fig. 3 ▸ *a*). Since the title compound is a coordination polymer, this analysis also includes the bonding information at the edge of the asymmetric unit. The overall two-dimensional fingerprint plot is depicted in Fig. 3 ▸ *b* decomposed into specific inter­actions. The central spike with the tip at (*d_i_*, *d_e_*) = (1.30, 1.41) directly represents the Cd—Br bond length with the relative contribution of 2.5%, while two other closely lying spikes with tips at (*d_i_*, *d_e_*) = (1.10, 1.30)/(1.30/1.10) correspond to the shorter Cd—N bond length with the contribution of 12.3%. The rest of the contacts belong to weak hydrogen bonds. At 37.5%, the largest contribution to the overall crystal packing is from Br⋯H/H⋯Br inter­actions, which form characteristic wings of the plot with tips at (*d_i_*, *d_e_*) = (0.90, 1.60)/(1.60/0.90). Other inter­actions, H⋯H (22.2%), H⋯C/C⋯H (9.3%) and H⋯N/N⋯H (10.6%), are mainly distributed in the middle part of the plot.

## 5. Database survey

A search of the Cambridge Structural Database (CSD version 5.43, update of November 2022; Groom *et al.*, 2016 ▸) reveals one hit with the 3-apz bridging ligand in a binuclear Cu^2+^ complex TIXDAH with oxalyl anions as coligands (Świtlicka-Olszewska *et al.*, 2014 ▸). In the complex, the same coordination mode of the ligand is observed, but with a shorter inter­metallic separation (4.583 Å) than in the title compound, which is due to the different chemical nature and square-pyramidal coordination geometry of the central ion.

## 6. Synthesis and crystallization

CdBr_2_·4H_2_O and 3-apz were purchased from Sigma Aldrich and were used without further purification. Colourless crystals were obtained by the reaction of 1 mmol of CdBr_2_·4H_2_O (344 mg) and 2 mmol of 3-apz (166 mg) in 10 ml of ethanol (96%). The reaction mixture was left overnight in an open vial, leading to the formation of crystals suitable for single-crystal X-ray analysis. Elemental analysis calculated for C_6_H_10_Br_2_CdN_6_: C, 16.44; H, 2.30; N, 19.17. Found: C, 16.56; H, 2.18; N, 19.33. IR (KBr; cm^−1^): 3321(*s*) ν(NH); 1592(*m*), 1554(*m*) and 1528(*s*) ν(C=N/C_3-apz_).

## 7. Refinement

Crystal data, data collection and structure refinement details are summarized in Table 2 ▸. H atoms were refined as riding [C—H = 0.83–0.92 Å with *U*_iso_(H) = 1.2*U*_eq_(C/N)].

## Supplementary Material

Crystal structure: contains datablock(s) I. DOI: 10.1107/S2056989023009751/tx2078sup1.cif

Structure factors: contains datablock(s) I. DOI: 10.1107/S2056989023009751/tx2078Isup2.hkl

Supporting information file. DOI: 10.1107/S2056989023009751/tx2078Isup3.cdx

CCDC reference: 2306409

Additional supporting information:  crystallographic information; 3D view; checkCIF report

## Figures and Tables

**Table 1:** Hydrogen-bond geometry (Å, °)

*D*—H⋯*A*	*D*—H	H⋯*A*	*D*⋯*A*	*D*—H⋯*A*
N2—H2⋯Br1^i^	0.86	2.80	3.377 (9)	126
N3—H3*A*⋯Br1^ii^	0.89	2.61	3.484 (9)	169
N3—H3*B*⋯Br1^iii^	0.89	2.79	3.640 (9)	160

Symmetry codes: (i) 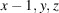 ; (ii) 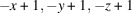 ; (iii) 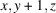 .

**Table 2:** Experimental details

Crystal data	
Chemical formula	[CdBr_2_(C_3_H_5_N_3_)_2_]
*M*_r_	438.42
Crystal system, space group	Triclinic, *P* 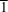
Temperature (K)	293
*a*, *b*, *c* (Å)	5.0515 (2), 6.7912 (3), 8.7083 (6)
α, β, γ (°)	83.585 (4), 79.907 (4), 86.833 (3)
*V* (Å^3^)	292.09 (3)
*Z*	1
Radiation type	Cu *K*α
μ (mm^−1^)	22.83
Crystal size (mm)	0.15 × 0.02 × 0.02

Data collection	
Diffractometer	XtaLAB Synergy, Dualflex, HyPix
Absorption correction	Multi-scan (*CrysAlis PRO*; Rigaku OD, 2020 ▸)
*T*_min_, *T*_max_	0.212, 1.000
No. of measured, independent and observed [*I* > 2σ(*I*)] reflections	5241, 1122, 1114
*R*_int_	0.036
(sin θ/λ)_max_ (Å^−1^)	0.631

Refinement	
*R*[*F* ^2^ > 2σ(*F* ^2^)], *wR*(*F* ^2^), *S*	0.045, 0.134, 1.27
No. of reflections	1122
No. of parameters	71
H-atom treatment	H-atom parameters constrained
Δρ_max_, Δρ_min_ (e Å^−3^)	0.94, −0.84

Computer programs: *CrysAlis PRO* (Rigaku OD, 2020 ▸), *SHELXT2018/2* (Sheldrick, 2015*a* ▸), *SHELXL2018/3* (Sheldrick, 2015*b* ▸) and *OLEX2* (Dolomanov *et al.*, 2009 ▸).

## supplementary crystallographic information

### Crystal data

**Table:**

[CdBr_2_(C_3_H_5_N_3_)_2_]	*Z* = 1
*M**_r_* = 438.42	*F*(000) = 206
Triclinic, *P*1	*D*_x_ = 2.492 Mg m^−^^3^
*a* = 5.0515 (2) Å	Cu *K*α radiation, λ = 1.54184 Å
*b* = 6.7912 (3) Å	Cell parameters from 4554 reflections
*c* = 8.7083 (6) Å	θ = 5.2–76.6°
α = 83.585 (4)°	µ = 22.83 mm^−^^1^
β = 79.907 (4)°	*T* = 293 K
γ = 86.833 (3)°	Needle, clear light colourless
*V* = 292.09 (3) Å^3^	0.15 × 0.02 × 0.02 mm

### Data collection

**Table:**

XtaLAB Synergy, Dualflex, HyPix diffractometer	1122 independent reflections
Radiation source: micro-focus sealed X-ray tube, PhotonJet (Cu) X-ray Source	1114 reflections with *I* > 2σ(*I*)
Mirror monochromator	*R*_int_ = 0.036
Detector resolution: 10.0000 pixels mm^-1^	θ_max_ = 76.8°, θ_min_ = 5.2°
ω scans	*h* = −6→6
Absorption correction: multi-scan (CrysAlisPro; Rigaku OD, 2020)	*k* = −8→8
*T*_min_ = 0.212, *T*_max_ = 1.000	*l* = −10→10
5241 measured reflections	

### Refinement

**Table:**

Refinement on *F*^2^	Hydrogen site location: inferred from neighbouring sites
Least-squares matrix: full	H-atom parameters constrained
*R*[*F*^2^ > 2σ(*F*^2^)] = 0.045	*w* = 1/[σ^2^(*F*_o_^2^) + (0.0297*P*)^2^ + 5.2025*P*] where *P* = (*F*_o_^2^ + 2*F*_c_^2^)/3
*wR*(*F*^2^) = 0.134	(Δ/σ)_max_ < 0.001
*S* = 1.27	Δρ_max_ = 0.94 e Å^−^^3^
1122 reflections	Δρ_min_ = −0.84 e Å^−^^3^
71 parameters	Extinction correction: *SHELXL2018/3* (Sheldrick, 2015b), Fc^*^=kFc[1+0.001xFc^2^λ^3^/sin(2θ)]^-1/4^
0 restraints	Extinction coefficient: 0.0028 (7)
Primary atom site location: dual	

### Special details

**Table:**

Geometry. All esds (except the esd in the dihedral angle between two l.s. planes) are estimated using the full covariance matrix. The cell esds are taken into account individually in the estimation of esds in distances, angles and torsion angles; correlations between esds in cell parameters are only used when they are defined by crystal symmetry. An approximate (isotropic) treatment of cell esds is used for estimating esds involving l.s. planes.

### Fractional atomic coordinates and isotropic or equivalent isotropic displacement parameters (Å^2^)

**Table:**

	*x*	*y*	*z*	*U*_iso_*/*U*_eq_	
Cd1	1.000000	0.500000	0.500000	0.0316 (4)	
Br1	0.9072 (2)	0.25426 (18)	0.28787 (13)	0.0413 (4)	
N2	0.3030 (18)	0.6427 (14)	0.1530 (10)	0.037 (2)	
H2	0.171982	0.587161	0.125640	0.044*	
N1	0.3397 (17)	0.6397 (13)	0.3036 (10)	0.0336 (19)	
N3	0.6733 (17)	0.7584 (13)	0.4288 (11)	0.0341 (19)	
H3A	0.539337	0.762302	0.509882	0.041*	
H3B	0.752934	0.874184	0.416228	0.041*	
C3	0.491 (2)	0.7406 (17)	0.0522 (14)	0.040 (2)	
H3	0.503093	0.760630	−0.056213	0.048*	
C2	0.664 (2)	0.8068 (16)	0.1397 (13)	0.036 (2)	
H2A	0.818189	0.878515	0.103040	0.043*	
C1	0.559 (2)	0.7436 (15)	0.2937 (12)	0.032 (2)	

### Atomic displacement parameters (Å^2^)

**Table:**

	*U*^11^	*U*^22^	*U*^33^	*U*^12^	*U*^13^	*U*^23^
Cd1	0.0268 (6)	0.0368 (6)	0.0324 (6)	−0.0077 (4)	−0.0069 (4)	−0.0032 (4)
Br1	0.0427 (7)	0.0449 (7)	0.0387 (7)	−0.0148 (5)	−0.0056 (5)	−0.0109 (5)
N2	0.031 (5)	0.048 (5)	0.032 (5)	−0.007 (4)	−0.008 (4)	−0.003 (4)
N1	0.026 (4)	0.040 (5)	0.036 (5)	−0.010 (4)	−0.004 (4)	−0.002 (4)
N3	0.030 (4)	0.033 (4)	0.043 (5)	−0.009 (4)	−0.014 (4)	−0.003 (4)
C3	0.040 (6)	0.042 (6)	0.038 (6)	−0.006 (5)	−0.009 (5)	0.001 (5)
C2	0.032 (5)	0.035 (6)	0.041 (6)	−0.007 (4)	−0.007 (5)	0.003 (4)
C1	0.031 (5)	0.035 (5)	0.032 (5)	0.000 (4)	−0.009 (4)	−0.002 (4)

### Geometric parameters (Å, º)

**Table:**

Cd1—Br1^i^	2.7379 (11)	N1—C1	1.332 (13)
Cd1—Br1	2.7379 (11)	N3—H3A	0.8900
Cd1—N1^ii^	2.358 (9)	N3—H3B	0.8900
Cd1—N1^iii^	2.358 (9)	N3—C1	1.413 (13)
Cd1—N3	2.446 (9)	C3—H3	0.9300
Cd1—N3^i^	2.446 (9)	C3—C2	1.380 (15)
N2—H2	0.8600	C2—H2A	0.9300
N2—N1	1.355 (12)	C2—C1	1.382 (15)
N2—C3	1.326 (15)		
			
Br1—Cd1—Br1^i^	180.0	N2—N1—Cd1^iv^	117.1 (6)
N1^iii^—Cd1—Br1^i^	92.6 (2)	C1—N1—Cd1^iv^	138.2 (7)
N1^ii^—Cd1—Br1	92.6 (2)	C1—N1—N2	104.1 (8)
N1^ii^—Cd1—Br1^i^	87.4 (2)	Cd1—N3—H3A	107.7
N1^iii^—Cd1—Br1	87.4 (2)	Cd1—N3—H3B	107.7
N1^ii^—Cd1—N1^iii^	180.0	H3A—N3—H3B	107.1
N1^iii^—Cd1—N3^i^	88.8 (3)	C1—N3—Cd1	118.3 (7)
N1^ii^—Cd1—N3	88.8 (3)	C1—N3—H3A	107.7
N1^ii^—Cd1—N3^i^	91.2 (3)	C1—N3—H3B	107.7
N1^iii^—Cd1—N3	91.2 (3)	N2—C3—H3	126.7
N3—Cd1—Br1^i^	85.2 (2)	N2—C3—C2	106.5 (10)
N3^i^—Cd1—Br1	85.2 (2)	C2—C3—H3	126.7
N3^i^—Cd1—Br1^i^	94.8 (2)	C3—C2—H2A	127.4
N3—Cd1—Br1	94.8 (2)	C3—C2—C1	105.1 (10)
N3^i^—Cd1—N3	180.0	C1—C2—H2A	127.4
N1—N2—H2	123.6	N1—C1—N3	120.3 (9)
C3—N2—H2	123.6	N1—C1—C2	111.5 (9)
C3—N2—N1	112.7 (9)	C2—C1—N3	127.8 (10)
			
Cd1^iv^—N1—C1—N3	14.7 (16)	N2—C3—C2—C1	−1.1 (13)
Cd1^iv^—N1—C1—C2	−172.3 (8)	N1—N2—C3—C2	0.1 (13)
Cd1—N3—C1—N1	87.2 (11)	C3—N2—N1—Cd1^iv^	173.9 (7)
Cd1—N3—C1—C2	−84.6 (12)	C3—N2—N1—C1	0.9 (12)
N2—N1—C1—N3	−174.6 (9)	C3—C2—C1—N1	1.7 (13)
N2—N1—C1—C2	−1.6 (12)	C3—C2—C1—N3	174.1 (10)

Symmetry codes: (i) −*x*+2, −*y*+1, −*z*+1; (ii) −*x*+1, −*y*+1, −*z*+1; (iii) *x*+1, *y*, *z*; (iv) *x*−1, *y*, *z*.

### Hydrogen-bond geometry (Å, º)

**Table:**

*D*—H···*A*	*D*—H	H···*A*	*D*···*A*	*D*—H···*A*
N2—H2···Br1^iv^	0.86	2.80	3.377 (9)	126
N3—H3*A*···Br1^ii^	0.89	2.61	3.484 (9)	169
N3—H3*B*···Br1^v^	0.89	2.79	3.640 (9)	160

Symmetry codes: (ii) −*x*+1, −*y*+1, −*z*+1; (iv) *x*−1, *y*, *z*; (v) *x*, *y*+1, *z*.

### Hydrogen-bond geometry (Å, °).

**Table:**

D—H···A	H···A	D···A	D—H···A
N2H···Br^i^	3.377 (1)	2.803 (1)	125.63 (1)
N3–H···Br^ii^	3.848 (1)	2.607 (1)	168.92 (1)
N3–H···Br^iii^	3.640 (1)	2.791 (1)	159.89 (1)

Symmetry codes: (i) 1+x,y,z; (ii) 1-x,1-y,1-z; (iii) x,1+y,z
